# Honored Local Names

**Published:** 1880-04

**Authors:** Ausburn Towner


### HONORED LOCAL NAMES.

**And Some Incidents Connected with the Lives of their Bearers.**

AUSBURN TOWNER.

Every county in the State has family names, that are peculiar to the locality, and are held in honor and respect by the public memory, whether the present wearers of them may be worthy or not. Their reputation may not extend beyond the limits of a township, but there, embalmed in the designation of a creek, a street or a village they live, gaining a stronger and stronger hold on the neighborhood, as years remove the period when their wearers lived further and further back in the past, and surround their hard pioneer lives, and their early struggles with nature in its wildest form, with romance and mystery. In Chemung county, the names of the Bennitts, the Baldwins, the Covells, the Maxwells, the Arnots and the Tuttles have the same relative social altitude as in New York, the names of the Astors, Livingstons, Stuyvesants and Bleeckers. The field they occupy is not so large, nor are the interests they represent so extensive, but their relations to their several communities are of a similar nature. Comfort Bennitt was, for many years, the largest landholder in this part of the State. He came early in life to this region, and living until he was more than ninety years of age, became the owner of nearly 2,500 acres of land. It was a rough, wild life that was lived in this neighborhood in his youth. On what is now one of the finest avenues in this city, it was the custom then, every Sunday afternoon, to have horse-races, the level ground and the straight road presenting a mile track of unsurpassed excellence. In the loss of one of his arms and one of his eyes, over which he wore a large green patch, Bennitt bore testimony, for more than sixty years, to the severity of the bitter personal contests that were waged in his youth, in which he bore a part. His large possessions testified too, to his close calculation and prudent living. Late in life, he was, what is called, "happily converted," and became a leading member of the church to which he attached himself. He was the father of a very large family, sons and daughters, but, as many as they were, he was able to settle each one in life with a large farm, well stocked and provided with comfortable buildings. His descendants are numerous, and all of them occupy conspicuous positions in the county, conveying the name honored and respected to posterity.

Hathaway is another memorable name in Chemung county, although it is fast fading away from the recollection of advancing generations. It does not deserve to do so, but he who wore it and made it distinguished in his day, left no descendants bearing his own name, for he did not marry, and there remain no monuments of his genius or industry to recall him to the memory. He held a commission under the old militia laws of the State, and was known far and wide as Col. Hathaway. By profession, he was a lawyer, after the old style of lawyers, absorbing rather than studying the law, and its bearings upon whatever case he might have on hand. He was a very handsome man in his younger days, and when member of Assembly from this county, attracted a great deal of attention in Albany. Gov. Bouck complimented him very highly, and prophesied for him a conspicuous career. He could have fulfilled the prediction. He was a friend of Prince John VanBuren, and was of the same political faith, the two men resembling each other in many particulars, in none perhaps more closely than their effective open air stump speaking. In this, Col. Hathaway was almost unapproachable, never failing to carry his audience with him in any direction that he might wish.

In one of the liveliest and most exciting political canvasses, Col. Hathaway was to speak in a neighbohood where he was well known. Some of his whig friends prepared a practical joke for him. One of them who had a particularly prepossessing wife, sent up to the Colonel, while he was in the midst of his speech, a neat parcel, with an intimation that it contained an American flag, with which he could produce a stunning effect by preparing a climax into which it would fit, and then suddenly unrolling it. The Colonel worked himself up to the desired point, and then tearing off the paper from the parcel, flung out before the amazed gaze of the great throng, not, as he supposed the banner of the stars and stripes, but a red petticoat! For an instant there was a hush in the assemblage, like the deathly calmness that precedes the simoon, and then came a roar of laughter, the echo of which seems yet to linger around the grove. In the midst of it all, Col. Hathaway was not the least embarrassed or abashed. He let the tumult before him tire itself out, while he was observed to be carefully examining the garment he continued to hold in his hand, and closely scrutinizing the paper in which it had been wrapped. After a time, the laughter subsided, and he quieted it with a gesture. Holding the petticoat toward the audience, with an air of much concern and assurance, he said :

"There is no name, that I can discover, sent with this garment, but I think I recognize it, nevertheless."

Another roar of laughter followed these rather pertinent words, and when it had subsided, Col. Hathaway went on with his speech, as if nothing had happened, but his whig friend, who had confided beforehand to a few intimates, his intended prank, never heard the last of it.

Gen. Whitney Gates is another name, well-known not many years ago in this county, but fast passing into forgetfulness. Whence his title, it would be hard to determine. He began his active life as a stage driver, when to follow that occupation elevated a man socially, as much as it lifted him among his fellow men. He became interested in time, in all the stage lines centering here, ran a large and flourishing livery stable at one time, and ended his life as the carrier of the mails between the railroad station and the Post Office. He was a protege of Col. Hathaway, and leaned heavily on him in all emergencies. He died only a few years ago.

Lowman is another conspicuous family name of the county. Jacob, the first of the name here, came to this valley seventy years ago, and settled on a farm in the township of Chemung, seven or eight miles east of this City. His youngest son, also named Jacob, a bachelor, nearly sixty years of age, is the chief representative of the family now, and is one of the largest landholders in the state, his farm or farms embracing about three thousand acres in the townships of Ashland, Baldwin and Chemung. Of all the land he holds, and it is all entirely unencumbered, he inherited only one hundred and seventy acres from his father. He began accumulating about thirty-five years ago, and will probably continue to increase his possessions as long as he lives. In addition to his own home farm of seven hundred acres, in the richest part of the Chemung valley, he has eight other farms that are worked on shares by tenants. The greatest product is butter, and Mr. Lowman has the reputation in New York city of sending to market, the largest supply that is delivered there from any one man. The quality too is as exceptional as the quantity. The average number of cows on his property is three hundred, and the average amount of butter from each cow, in a season, is two firkins of eighty pounds each, making in all, about fifty-thousand pounds of butter in a season. Mr. Lowman raises all his own stock, prefering for his purposes a cow of Alderney and Durham, with no serious objection to a little of the Devon blood. He breeds his own horses too, never, as he said recently, having bought but one horse in his life. He has about eighty horses on his property and about one hundred head of young cattle. His cows last on an average, ten years apiece.

His other farm products are, in proportion, as large as his butter and are probably equalled by no farmer in the State. Last year he raised twenty-five hundred bushels of wheat and five hundred bushels of barley to sell. The coarse grains, corn and oats, that he raises, are all used on his farms. He also raises large quantities of tobacco, and in its cultivation, discovered a manure that largely increased the yield, this being one part taken from his large hennery; two parts common wood ashes, and one part plaster. The difference in the plants between those on which this combination was applied, and those on which it was not, could readily be seen. One leaf taken from the field and lying now before the writer, measures more than three feet from its point to where it was cut from the stem.

There is a little village on the property, called, in the Post Office Directory, "Lowmans." It has a saw mill, a grist mill, a store and half a dozen or more dwelling houses, all owned by Mr. Lowman, and although it is somewhat removed from the great thoroughfares it has telegraphic communication with the outside world, and a morning and evening mail. Mr. Lowman's farm life is a phase of existence that has few examples in this state, at least, so far as the magnitude of his possessions and operations is concerned.

Hoffman is another Chemung county name, that, not only in memory of the worth of the original bearer of it here, but because of those who now honor it, is entitled to great respect and consideration. William Hoffman, the first representative of the name in this county, was a soldier in the war of 1812, and settled here soon after that time. His sons have all held conspicuous social and political positions in the county. The youngest, Col. H. C. Hoffman, called "Barney," because of a boyishly acquired nickname, now our Member of Assembly, at the close of the civil war, in 1864, established the first "Creamery" ever built in this state or elsewhere. He discovered an extraordinary spring about seven miles north of this city, and over it built his creamery. It was not looked upon with much favor by the farmers, at first, but now he buys the milk from fifty-four dairies, averaging in the height of the season, between seven thousand and eight thousand quarts, and making annually thirty-five hundred cheeses, and between forty and fifty tons of butter. Much of his cheese is sent to England, where it finds a ready market.

Chemung county butter and Chemung county tobacco command higher prices than the tobacco and butter from any other part of the country.

There have been some changes in the names of families in Chemung county, that are rather curious. They remind one somewhat of the study of philologists who make out the celebrated Chinese philosopher Fo to be identical with Noah, in this wise: Take the N and the final ah from the patriarch's name and add an F, which in many languages is interchangeable with N, and there you have it ; not only the the identical person, but the identical name. How the name of Buckhorn can be eliminated down to Bacon, or Vanderwerker down to Bogart, might puzzle even the most learned philologist; but the records of this county show that it has been done.

Take the first name. Buckhorn became, from poor chirography, or bad pronunciation, Beckhorn. In the same way, it easily became Backhorn. The k might well be deemed superfluous and from Bachorn to Bacon is an exceedingly short step. It has been taken in this county in more than one instance.

And Vanderwerker. That presents more astonishing difficulties. For some reason or other, perhaps because in the original language werker and bogart meant the same, the name was changed from Vanderwerker to Vanderbogart. The change became known by reason of the search for a deed that was supposed to have been lost. It belonged to Mr. Vanderbogart, but the property had been conveyed to an immediate ancester whose name was Vanderwerker. In a generation or so Vanderbogart became Vanbogart and soon after, the Van was lopped off and the name became simply Bogart.

Sometimes, the change is made by simply translating, as Zimmerman to Carpenter, each a common name in its respective tongue. The family name of Fairfield, common in many localities in this country, was originally the same in French, Beauchamp. There was a universal translation from the original to the English tongue, some fifty or seventy-five years ago, when a conspicuous member of the family in Kentucky was hanged for a most atrocious and unnecessary murder.

